# An *In Vitro* Comparison of Anti-Tumoral Potential of Wharton’s Jelly and Bone Marrow Mesenchymal Stem Cells Exhibited by Cell Cycle Arrest in Glioma Cells (U87MG)

**DOI:** 10.3389/pore.2021.584710

**Published:** 2021-04-08

**Authors:** Nazneen Aslam, Elham Abusharieh, Duaa Abuarqoub, Dana Alhattab, Hanan Jafar, Walhan Alshaer, Razan J. Masad, Abdalla S. Awidi

**Affiliations:** ^1^ Cell Therapy Center, The University of Jordan, Amman, Jordan; ^2^ Department of Pharmaceutical science, Faculty of Pharmacy, The University of Jordan, Amman, Jordan; ^3^ Department of Pharmacology and Biomedical Sciences, Faculty of Pharmacy and Medical Sciences, University of Petra. Amman, Jordan; ^4^ Laboratory for Nanomedicine, Division of Biological & Environmental Science & Engineering (BESE), King Abdullah University of Science and Technology (KAUST), Thuwal, Saudi Arabia; ^5^ Department of Anatomy and Histology, School of Medicine, The University of Jordan, Amman, Jordan; ^6^ Department of Medicine, School of Medicine, The University of Jordan, Amman, Jordan; ^7^ Department of Hematology and Oncology, Jordan University Hospital, The University of Jordan, Amman, Jordan; ^8^ Department of Hematology and Oncology, The University of Jordan, Amman, Jordan

**Keywords:** mesenchymal stem cells, glioblastoma, secretome, WJ-MSCs, BM-MSC, cell lysate MSC-condition media

## Abstract

The therapeutic potential of mesenchymal stem cells (MSCs) for various malignancies is currently under investigation due to their unique properties. However, many discrepancies regarding their anti-tumoral or pro-tumoral properties have raised uncertainty about their application for anti-cancer therapies. To investigate, if the anti-tumoral or pro-tumoral properties are subjective to the type of MSCs under different experimental conditions we set out these experiments. Three treatments namely cell lysates (CL), serum-free conditioned media and FBS conditioned media (FBSCM) from each of Wharton’s Jelly MSCs and Bone Marrow-MSCs were applied to evaluate the anti-tumoral or pro-tumoral effect on the glioma cells (U87MG). The functional analysis included; Morphological evaluation, proliferation and migration potential, cell cycle analysis, and apoptosis for glioma cells. The fibroblast cell line was added to investigate the stimulatory or inhibitory effect of treatments on the proliferation of the normal cell. We found that cell lysates induced a generalized inhibitory effect on the proliferation of the glioma cells and the fibroblasts from both types of MSCs. Similarly, both types of conditioned media from two types of MSCs exerted the same inhibitory effect on the proliferation of the glioma cells. However, the effect of two types of conditioned media on the proliferation of fibroblasts was stimulatory from BM-MSCs and variable from WJ-MSCs. Moreover, all three treatments exerted a likewise inhibitory effect on the migration potential of the glioma cells. Furthermore, we found that the cell cycle was arrested significantly at the G1 phase after treating cells with conditioned media which may have led to inhibit the proliferative and migratory abilities of the glioma cells (U87MG). We conclude that cell extracts of MSCs in the form of secretome can induce specific anti-tumoral properties in serum-free conditions for the glioma cells particularly the WJ-MSCs and the effect is mediated by the cell cycle arrest at the G1 phase.

## Introduction

Glioblastoma Multiforme (GBM) is the most aggressive brain tumor that is characterized by the rapid growth, invasiveness, and poor prognosis due to many factors including drug resistance, difficulty to repair damaged brain cells, and the inability of many drugs to cross the blood-brain barrier [1]. Despite multimodal novel treatment options for GBM, the median survival time is still less than 15 months [2]. Currently, many new agents are under investigation to target the cell function and signaling pathways in cancers. Among them are included: Protein kinases, angiogenesis inhibitors, monoclonal antibodies, methyltransferase inhibitors, small interfering RNAs, and mesenchymal stem cells (MSCs) [3].

The therapeutic potential of MSCs in regenerative medicine is relatively well-established but the anti-tumorigenic effect of MSCs has received attention recently. The interest in MSCs for cell therapy is justified by their unique properties [4]. Given their attributes, MSCs are under evaluation in several clinical trials (http://www.clinicaltrials.gov/ct2/results?term = MSC) [5]. Due to the specific homing ability of MSCs to tumor sites in humans, they could be used as delivery vehicles for targeted anticancer drugs or gene-therapy [6–10]. However, this putative approach raised many safety concerns. Firstly, MSCs have intrinsic anti-tumorigenic activities but they also hold pro-tumorigenic properties while suppressing the immune system and modifying the micro-environment for tumor propagation [11]. Secondly, MSCs hold the ability to differentiate which has raised the possibility of their conversion to cancer cells in the tumor microenvironment [12, 13]. Additionally, in the case of allogenic transplantation, MSCs may confront the host immune system which can result in phagocytosis of the transplanted MSCs by immune cells [12, 13]. Moreover, the phenomenon of cannibalism of MSCs by cancer cells, resulting in the dormancy of tumor cells, has been presented as one of the possible reasons for tumor relapse [14]. Furthermore, the delivery of genetic material either by viral or non-viral vectors can increase the chance for induction of insertional mutations in MSCs which have raised the possibility of targeting of the normal cells by MSCs rather than the tumor cells [13, 15]. Therefore, further studies should be done to predispose the MSCs for clinical use in therapy for different types of cancers [16].

The mechanism by which MSCs favor multiple unrelated pathologies remains to be defined. Cell replacement was initially considered their main mechanism of action [17–19]. However, the lack of clear data about the MSCs engraftment at target organs and *trans*-differentiation [20] led the search for alternative mechanisms. Some experimental studies have reported that the regenerative potential of MSCs is attributed to the paracrine factors and not to their engraftment [21, 22]. This intriguing hypothesis has changed the therapeutic perspectives to develop cell-free strategies based on the use of MSCs secretome as evidenced by various diseases [23, 24]. Therefore, the conditioned media (CM) of MSCs or their purified extracellular vesicles can be used for cancer therapy instead of the injections of MSCs [25]. Very few studies using CM reported the growth factor levels and for most of them, the molecular data are also lacking [26–34]. It has been reported that the same type of cells yielded different growth factor levels relative to cell number, culture medium and condition, and CM processing [27]. That might be the core reason for discrepancies regarding the effect of CM from different sources of MSCs.

Anti or pro-tumorigenic behaviors of MSCs are attributed to types of cells derived from different sources and residing in different tumor environments. Wharton's Jelly/umbilical cord (UC-MSCs/WJ-MSCs) has shown more anti-cancer properties in different glioma cells and solid tumor cell lines [15]. Similarly, adipose tissue-MSCs (AT-MSCs) inhibited the proliferation and induced apoptosis of hepatic cancer cells [35], breast cancer cells [36], prostate cancer cells [37], and melanoma [38]. On the contrary, BM-MSCs have shown both, pro-tumorigenic and anti-proliferative activity on tumor growth [39, 40]. Probably, these conflicting effects of MSCs can be attributed to the experimental settings in different *in vitro* and *in vivo* models [41].

Cancer vaccines comprised of cancer cell lysates are currently under investigation for the generation of antitumor immune response for personalized medicine [15]. Similarly, it has been shown that neurotrophic factors in CM could access affected neurons in the central nervous system (CNS) by either directly crossing the blood-brain barrier or through the retrograde transport mechanism in CNS. CM from MSCs has already been applied for the treatment of many neurodegenerative diseases [42, 43], therefore CM might have a therapeutic effect for gliomas too as a combinatorial therapy. It has been reported that cell lysates and conditioned media from WJ-MSCs can induce inhibition of proliferation in a few cancer cell lines [3, 44]. In another report, conflicting results reported the initial inhibition with simultaneous proliferation with WJ-MSCs’ conditioned media in gliomas [45]. However, cell lysates and conditioned media of MSCs generated under the same culture conditions have not been tested on glioblastoma cell lines which is one of the aggressive cancers with poor prognosis.

To investigate if the conflicting (anti-tumoral or pro-tumoral) effects from MSCs are due to; the type of MSCs, or the time duration for the effect of trophic factors generated under the same culture conditions, we planned this study. We evaluated the anti-tumoral or pro-tumoral effect of the paracrine factors of WJ-MSCs and BM-MSCs in the form of cell extracts such as cell lysate and conditioned media mainly for the proliferation of glioma cells and subsequently for the morphology, and migration potential of the glioma cell line (U87MG). Conditioned media (FBSCM, SFCM) and cell lysates (CL), hereby named as treatments, were generated under the same culture conditions and were applied for the same time points (maximum 120 HR). The fibroblast cell line was added to investigate the effect of treatments on the proliferation (only) of normal cells.

## Materials and Methods

All procedures performed in this study were under the Declaration of Helsinki.

### Cell Culture

#### Generation of a Monolayer of U87MG

Cell line U87MG (U87MG ATCC-HTB14TM) was expanded according to the protocol described in [46] with a slight modification. Briefly, the cell line was expanded in standard culture media {DMEM-F121:1, L-Glutamine200 mM, 10,000 U/mL penicillin/streptomycin, 25 μg/ml amphotericin B, and 10% heat-inactivated Fetal Bovine Serum-GIBCO/Invitrogen Corporation, United States. The cells were seeded at 1 × 10^4^cells/cm^2^ in T75 cultures flasks and maintained at 37°C and 5% CO2. The culture medium was exchanged every 2–3 days.

#### Cultures of Mesenchymal Stem Cells and Fibroblasts Cell Lines

Cell lines of Wharton’s Jelly mesenchymal stem cells, WJ-MSCs (WJ1, WJ2, WJ3), bone marrow mesenchymal stem cells BM-MSC (BM1, BM2, BM3), and Human neonatal foreskin fibroblasts (DF1) were provided from The Cell Therapy Centre, University of Jordan. These cell lines were generated and maintained according to established protocols for the generation of MSCs [47] and fibroblasts [48]. Adherent cells were passaged when they reached 80% confluence. Cells at passage 3 were used for harvesting of conditioned media and cell lysates from two types of MSCs. Neonatal foreskin fibroblasts (DF1) were maintained in standard culture media (DMEM-F12 + 10%FBS) same as used for the generation of U87MG cells. Fibroblasts at passage 3 were used for MTT experiments only.

### Characterization of BM-Mesenchymal Stem Cells and WJ-Mesenchymal Stem Cells by FACS Analysis

For mesenchymal stem cell surface marker identification (MSCs), WJ-MSCs and BM-MSCs at passage 3 were harvested, stained, and analyzed for CD73, CD105, CD90, CD45, CD34, CD14, CD11b, and HLA-DR expression using the mesenchymal stem cell characterization kit (BD Biosciences, United States). Appropriate IgG isotype-matched antibodies and unstained cells were used as controls. Dead cells were excluded by adding 7-aminoactinomycin D (7-AAD; BD Bioscience, United States) before analysis. After staining, cells were acquired by FACS Canto II flow cytometer (BD Biosciences, United States) and analyzed by FACS Diva software (BD Biosciences, United States).

### Multi-Lineage Differentiation Potential of Mesenchymal Stem Cells

The multi-lineage potential was assessed by evaluating the ability of WJ-MSCs and BM-MSCs to differentiate into adipogenic, osteogenic, and chondrogenic lineages according to the protocol described [49]. Differentiation potential was performed using StemPro® Adipogenesis, Osteogenesis, and chondrogenesis differentiation kit (GIBCO, United States) according to the manufacturer's instructions. To detect adipogenic, osteogenic, and chondrogenic differentiation; oil red O stain, alizarin red S, and Alcian Blue stain were used, respectively.

### Preparation of Mesenchymal Stem Cells Extracts (Conditioned Media and Cell Lysates)

#### Harvest of Conditioned Media

MSCs from WJ-MSCs and BM-MSCs (P3) were cultured at the seeding density of 5 × 103cells/cm2 in MEM-alpha with 5% platelet lysate until cells were approximately 80–90% confluent. Then cells were washed twice with PBS and once with DMEM-F12 serum-free media. Next cells were conditioned in medium with serum (DMEM-F12 + 10% FBS as FBSCM) and without serum (DMEM-F12 only as SFCM). After 48HR, conditioned media (CM) were harvested, centrifuged at 300xg for 5 min, and filtered through a 0.22 µm syringe filter. To avoid frequent pH changes, CM was aliquoted and conserved at −80ºC until further use. For each experiment fresh aliquot was used. Two dilutions (50% with fresh culture media and 100%CM) from each type of CM (SFCM and FBSCM) were selected to investigate an inhibitory effect at different time points (24HR, 48HR, 72HR, 96HR, and 120HR).

#### Cell Lysates Preparation

Cell lysates were prepared using the Mammalian Cell Extraction Kit (ab65399) following the manufacturer's instruction. Briefly, after collection of conditioned media, attached cell layers were washed once with DPBS (Euroclone), trypsinized (0.25% trypsin, Invitrogen, United States), and centrifuged at 600xg for 5 min to obtain a cell pellet. The cell pellet was resuspended in 100 µL of cell lysis buffer, mixed well several times, and incubated on ice for 15–20 min followed by vortexing for 5 s. The contents were centrifuged in a microcentrifuge at 15,000xg for 5 min and the clear supernatant was collected while the pellet was discarded. Cell lysates were stored at −80°C until further use. Protein concentration was measured using the NanodropTM spectrophotometer (Nanodrop Technologies, United States).

### Functional Assays

#### Preliminary Experiments (MTT Assay) for Cell Lysates Concentrations

Initially different concentrations of cell lysate (5, 10, 15, and 20 μg/ml) from one sample of WJ-MSCs (WJ1) were tested and 20 μg/ml in fresh culture media (DMEM-F12 + 10%FBS) was selected for further use in all experiments).

#### Morphological Changes in Glioma Cells

Glioma cells from the U87MG cell line were cultured in six-well plates (TPP, United States) at the seeding density of 5 × 10^3^ cells/cm^2^ in standard culture media for the initial 24HR. The next day, the media were exchanged with treatments, cell lysate (20 μg/ml), FBSCM (100% and 50%), and SFCM (100% and 50%). The experiment was conducted for four days (96HR) with an exchange of fresh media (treatment) every 48HR. The changes in the cell morphology were recorded daily and photographed using an inverted phase-contrast microscope (Zeiss, Germany). Cells growing in standard culture media and serum-free media were kept as controls.

#### Cell Proliferation Assay (MTT) of Glioma Cells and Fibroblasts

The proliferation rates of the glioma cell line (U87MG) and human foreskin fibroblast cell line (DF1) were evaluated by MTT assay. In brief, the glioma cells were cultured at a seeding density of 5 × 10^3^/cm^2^ in 100 µL of culture media in 96 well plates (TPP, Sigma- Aldrich, United States). After 24HR the media were exchanged with treatments (Cell lysate, 100% CM, and 50% CM). Standard culture media and serum-free media were kept as controls. Media were exchanged every 48HR with respective treatment. The proliferation rate was determined at 24HR, 48HR, 72HR, 96HR and, 120HR by the reduction of (3-(4, 5-Dimethylthiazol-2-yl)-2,5-Diphenyltetrazolium Bromide) (MTT) solution according to the manufacturer's instruction. Optical density was measured at 560 nm using a microplate reader (GloMax®-multi, Promega, United States). Each proliferation (MTT) experiment was repeated three times (*n* = 3). Serum-free media (SFM as DMEM-F12 only) was kept as a control for SFCM and FBS media (DMEM-F12 + 10%FBS) was kept as a control for cell lysate and FBSCM. Glioma cells (U87MG) at passages 3-5 were used for all experiments. Similarly, fibroblasts (P3) were cultured at the same seeding density as glioma cells and after 24HR the media were exchanged with treatments {(Cell lysates and 100%CM only (SFCM, FBSCM)} from two types of MSCs. The rest of the procedure for MTT was being followed as described above for glioma cells.

**Note**. At this point, we found that cell lysates induced a generalized inhibitory effect on cancer cells and fibroblasts. While conditioned media induced a variable effect on fibroblasts. Therefore, further experiments were performed only with 100% CM since CM exhibited a selected effect on cancer cells.

#### Cell Cycle Analysis With Conditioned Media

The glioma cells (U87MG) were cultured at a seeding density of 1 × 10^4^/cm^2^ in 6 well plates (TPP. Sigma-Aldrich) for 24HR. Next, the culture media were exchanged with treatments (100%SFCM and 100%FBSCM). The experiment was conducted for 96HR with the exchange of fresh treatment every 48HR and cell cycle analysis was performed at 96HR time point. Cells growing in standard culture media and serum-free media were kept as controls. Cell cycle analysis for glioma cells treated with two types of CM was performed with PI staining according to the manufacturer’s instructions (Lifesciences). Canto BD II flow cytometer instrument (BD, United States) was used for running the samples. The experiment was conducted from six biological samples twice (*n = 2*) and the results are provided as averages of two independent experiments.

#### Apoptosis Using Annexin V and 7AAD for Conditioned Media Treated Glioma Cells

U87MG glioma cells were plated in 6-well plates (TPP.Sigma-Aldrich) at the seeding density of 1 × 10^4^/cm^2^ for 24HR. Next, the medium was replaced with SFCM and FBSCM from two types of MSCs, and induction was carried out for 96 HR. Annexin V/7AAD dyes were used according to manufacturer’s instructions (BD Biosciences). After incubation, the cells were analyzed by flow cytometer (FACS Canto II BD Biosciences).

#### Cell Migration Assay

U87MG glioma cells were cultured in cell inserts (Culture-Inserts2well.Ibidi, Germany) at the seeding density of 2 × 103/chamber in standard culture media until the glioma cells become 90% confluent. The inserts were removed and cells were serum-starved for 24HR. After that, the treatments (cell lysate, 100%SFCM, 100% FBSCM) were added and the experiment was conducted for 48HR thrice (*n = 3*). The closure of the scratch was photographed by using a phase-contrast microscope (Zeiss, Germany) at 0HR, 24HR, and 48HR, and ImageJ was used for analysis.

### Statistical Analysis

Data were analyzed using Microsoft Excel and GraphPrism software. Quantitative data were expressed as mean ± standard deviation. Data were evaluated by two-way ANOVA and Dunnet's post-test was used to analyze multiple comparisons (*p < 0.05).

## Results

### Immunophenotyping and Multi-Lineage Potential of Mesenchymal Stem Cells

**FIGURE 1:**
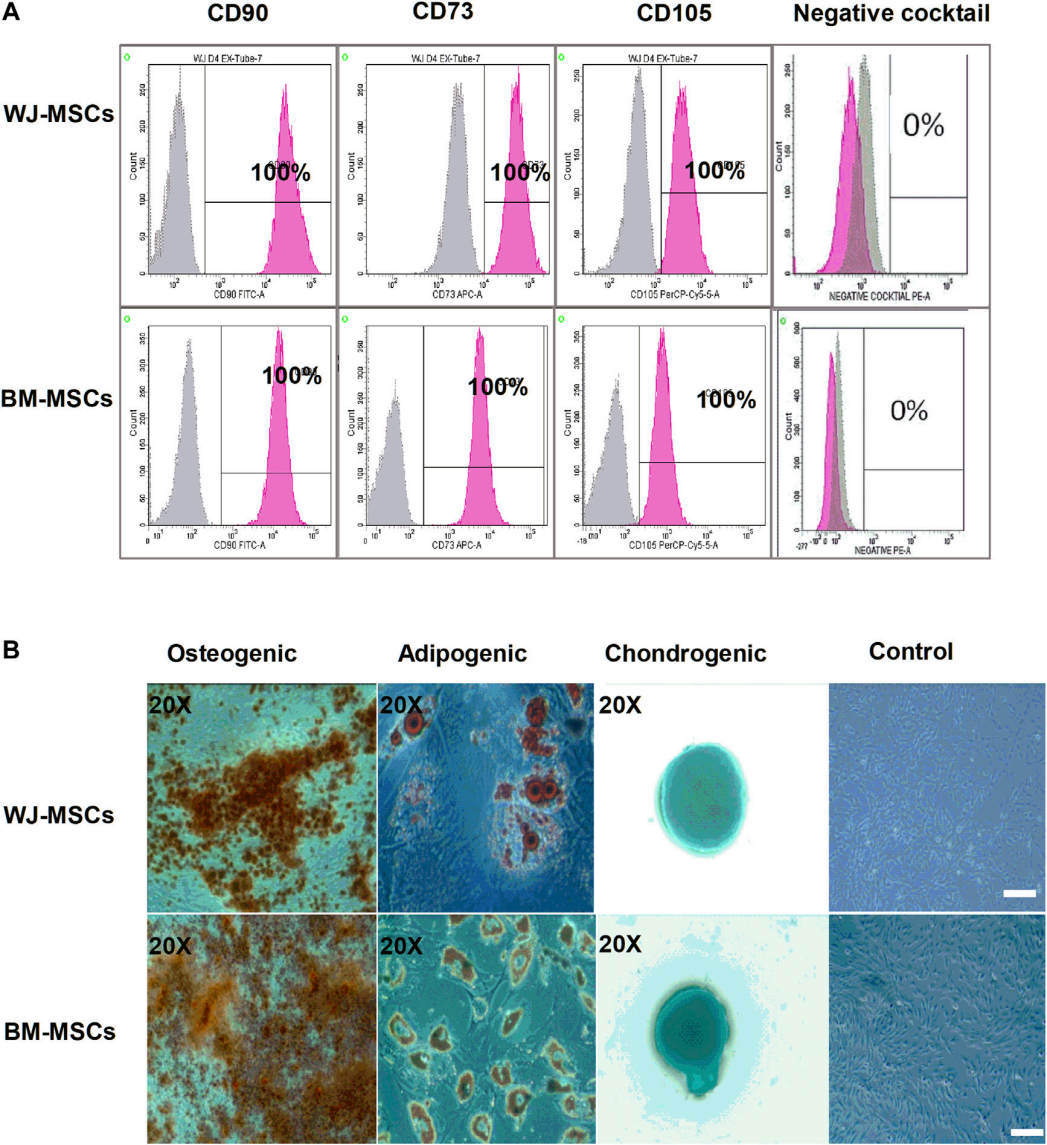
Characterization of mesenchymal stem cells (MSCs) derived from bone marrow (BM) and Wharton Jelly (WJ). **(A)** Flow cytometric histogram shows that cells are positive for the following markers: CD90, CD73, and CD105 and negative for expression of the negative cocktail markers including CD45, CD34, CD11b, and HLA-DR. **(B)** Microscopic photographs of tri-lineage differentiation potential of WJ-MSCs induced by Osteogenic (20X), adipogenic (20X), and chondrogenic (20X) differentiation compared to the undifferentiated cells as control (scale bar100 µm).

Surface marker expression for MSCs revealed the presence of positive and absence of negative markers (Figure 1A). The multi-lineage potential was also evaluated for osteogenesis, adipogenesis, and chondrogenesis. (Figure 1B). All samples of BM-MSCs (BM1, BM2, BM3) and WJ-MSCs (WJ1, WJ2, WJ3) successfully induced tri-lineage differentiation.

### Preliminary Results (MTT Assay) for Cell Lysates

**FIGURE 2:**
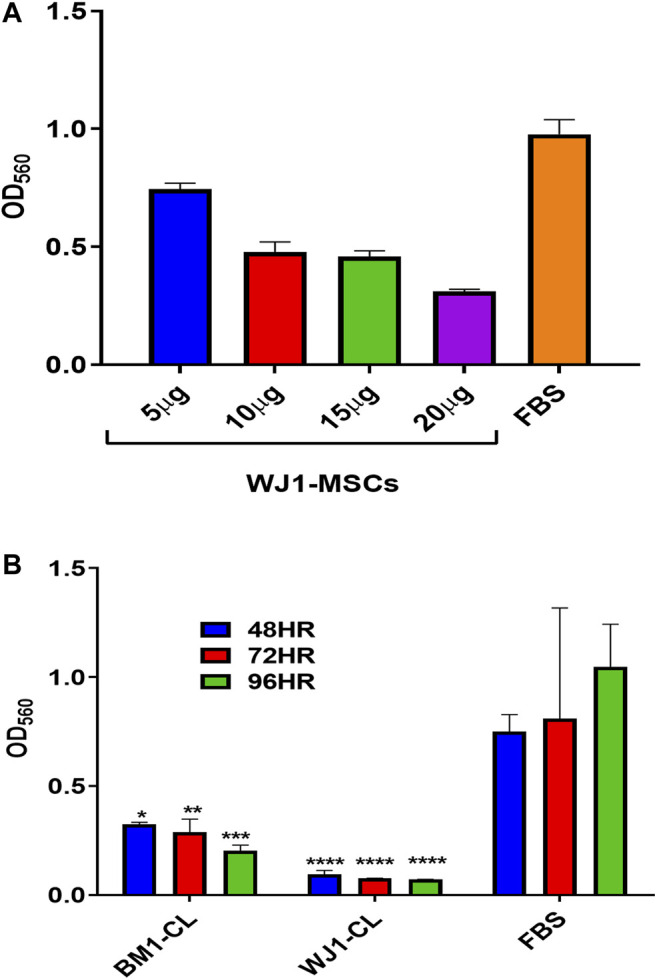
Preliminary data for the effect of cell lysate (CL) on the proliferation of glioma cells (U87MG). **(A)** Effect of different concentrations of WJ cell lysate on the proliferation of the glioma cells for 48HR. **(B)** Effect of cell lysate (20 μg/ml) from one of each of BM-MSCs and WJ-MSCs on the proliferation of glioma cells (*p < 0.05, **p < 0.01, ***p < 0.001, ****p < 0.0001) at 48,72, and 96HR.

Figure 1 summarized the results for the optimization of concentrations of cell lysate from one sample of BM1-MSCs and WJ1-MSCs. Cell lysates at 20 μg/ml in standard cultured media (DMEM-F12 + 10%FBS) showed an inhibitory effect at all-time points from each sample of BM1-MSC and WJ1-MSCs (Figures 2A,B).

### Cell Lysate Induced Vacuolation, Reduced Neurite Growths, and Inhibited Proliferation of the Glioma Cells (U87MG)

**FIGURE 3:**
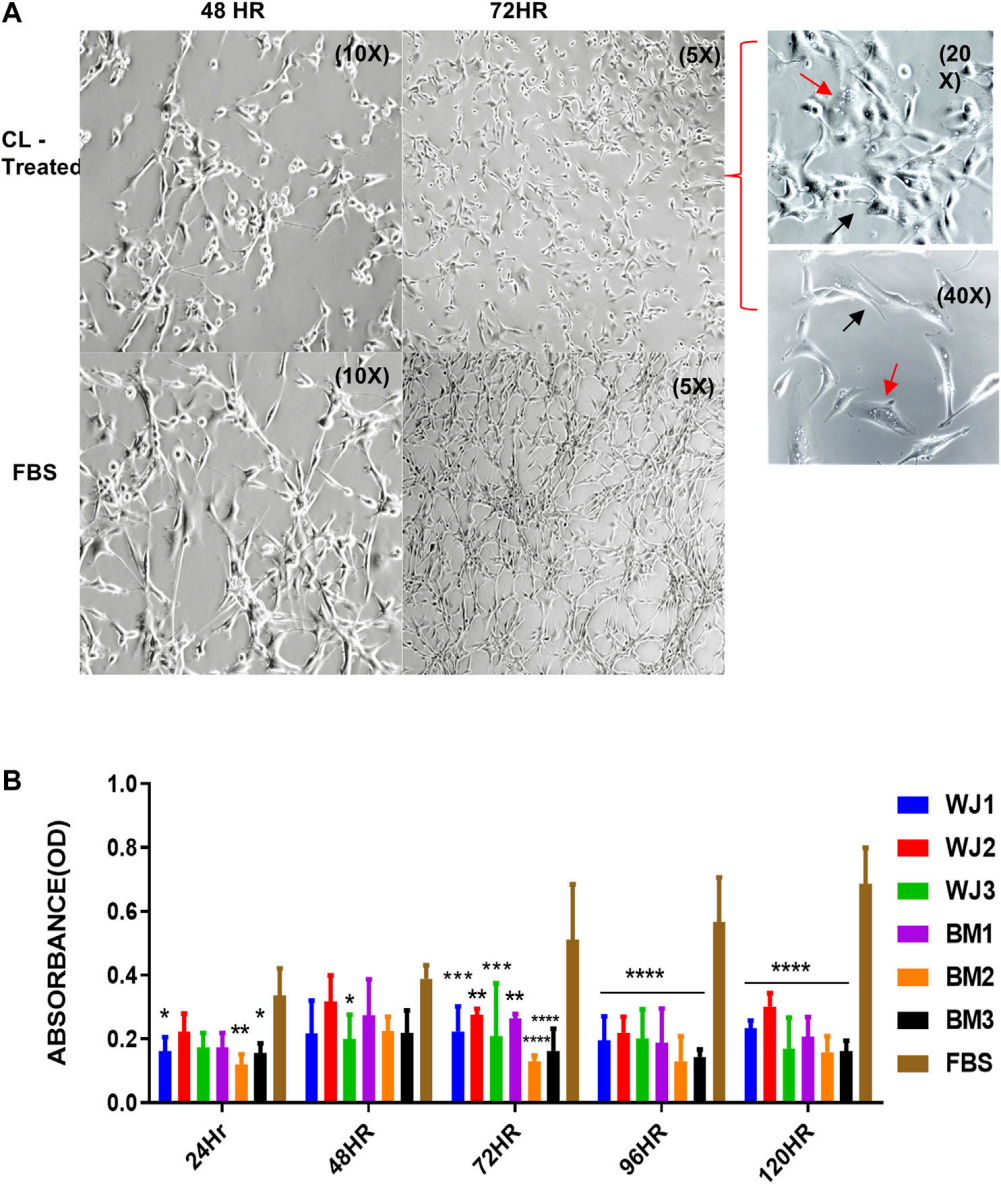
Effect of cell lysates from two types of mesenchymal stem cells on the morphology and proliferation of glioma cell line (U87MG). **(A)** Representative images of the morphological changes with cell lysates at 48HR (10X) and 72HR (5X) as compared to control FBS (10X, 5X). Glioma cells' neurite growths were reduced (black arrow) and vacuoles appeared (red arrow) as shown in treated cells at higher magnification of 20X and 40X. **(B)** Cell lysate from both types of MSCs significantly inhibited the proliferation of glioma cells with control FBS (*p < 0.05***p < 0.0,01, ****p < 0.0001).

U87MG cells were cultured at a minimal seeding density of 5 × 10^3^/cm^2^ to have a uniform cell morphology and to avoid cluster formation. We found that cell lysates changed the morphology of adherent layers as early as 48HR as compared to FBS (Figure 3A). The change in morphology was evident by the appearance of expanded shapes and vacuoles (red arrows) in cells indicating intracellular degradation, autophagy, or senescence. Their cell to cell communication was also halted as visible by the reduction of neurite growths (black arrows) (Figure 3A 72HR, 20x and 40X) as compared to control FBS. Based on microscopic evaluation it was found that the shortening of the neurites was consistent in the entire well whereas approximately 90% of the cells showed vacuolation at 72HR. Similarly, we found statistically significant and consistent inhibition of proliferation of the glioma cells from cell lysates after 48HR as compared to control FBS (Figure 3B). Consistent with preliminary results, cell lysates significantly inhibited the proliferative ability of the glioma cells (U87MG) from two types of MSCs.

### Conditioned Media (FBS Conditioned Media, Serum-Free Conditioned Media) Changed the Morphology, Inhibited Proliferation, and Arrested Cell Cycle at the G1 Phase of the Glioma Cells (U87MG)

#### Morphology of Glioma Cells

**FIGURE 4:**
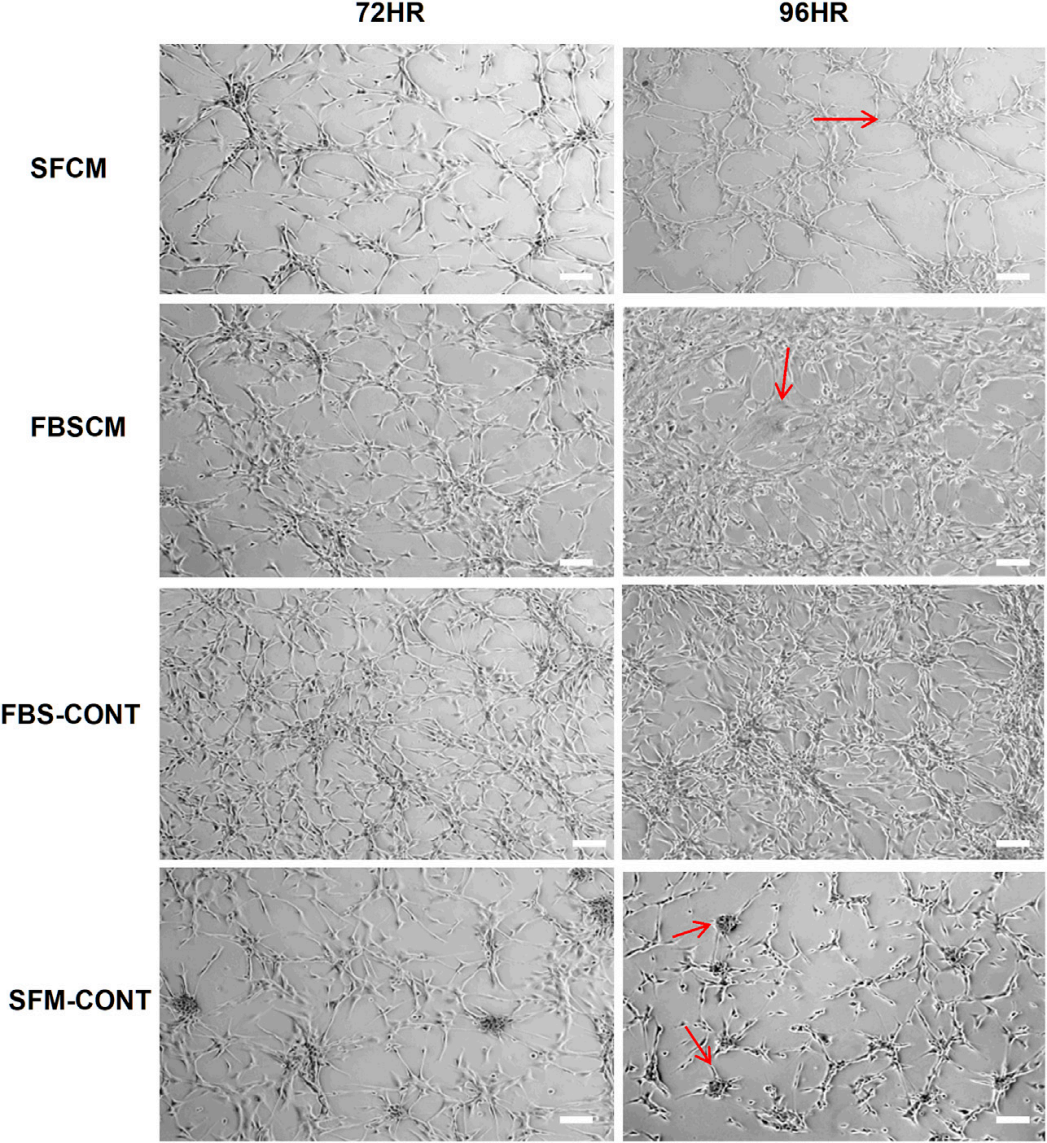
Representative images of the morphological changes with two types of conditioned media, SFCM and FBSCM, on glioma cells at 96HR. Arrows show the changes in cell shapes that happened in treated cells. It was noted that cells were expanded with the possibility of differentiation with both types of CM as compared to both controls (arrows). Interestingly cells in SFM control started forming spheres (arrows) whereas, treated cells with SFCM did not show this feature emphasizing the inhibitory effect of paracrine factors of MSCs on the stemness property of glioma cells (scale bar 100 µm).

The results of the morphological changes with CM treatments are summarized in Figure 4. We found that a change in morphological appearance from two types of CM (100%) was evident at 96HR (arrows). From FBSCM, cells became more expanded either like differentiated cells or senescent cells, rather than retaining mesh network and star-shaped morphology depicted by cells in control (FBS). Similarly, from SFCM, apart from expanded shapes, cells did not show sphere formation as shown by SFM control (Figure 4 arrows). Sphere formation in SFM control could be due to the presence of stemness in the cell line. However, we did not find any vacuolation in the glioma cells from both types of CM as seen by cell lysates. Moreover, we did not find any obvious morphological change with 50% CM (data not shown).

#### Proliferation of Glioma Cells

**FIGURE 5:**
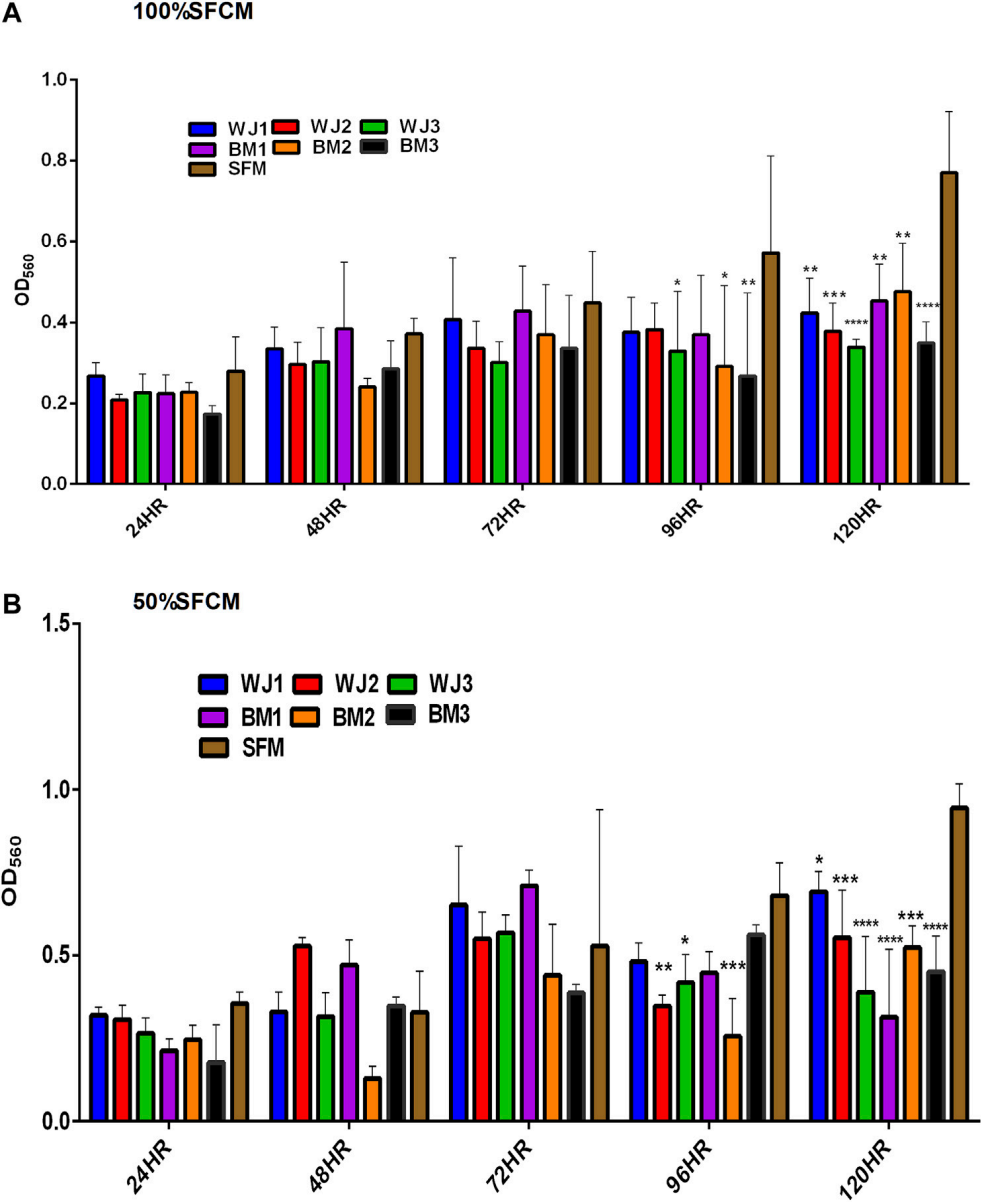
Evaluation of the effect of two concentrations of SFCM (100%, 50%) on the proliferation of glioma cells (U87MG) at different time points. **(A)** 100% SFCM. **(B)** 50% SFCM. It was noted that a significant inhibitory effect was evident at 96 and 120HR from both concentrations of SFCM. Regarding this, some samples consistently showed the same effect at both time points such as WJ3 and BM3 signifying some individual effects of MSCs while all the samples showed significant inhibition at 120HR (*p < 0.05, **p < 0.01, ***p < 0.001, ****p < 0.0001).

**FIGURE 6:**
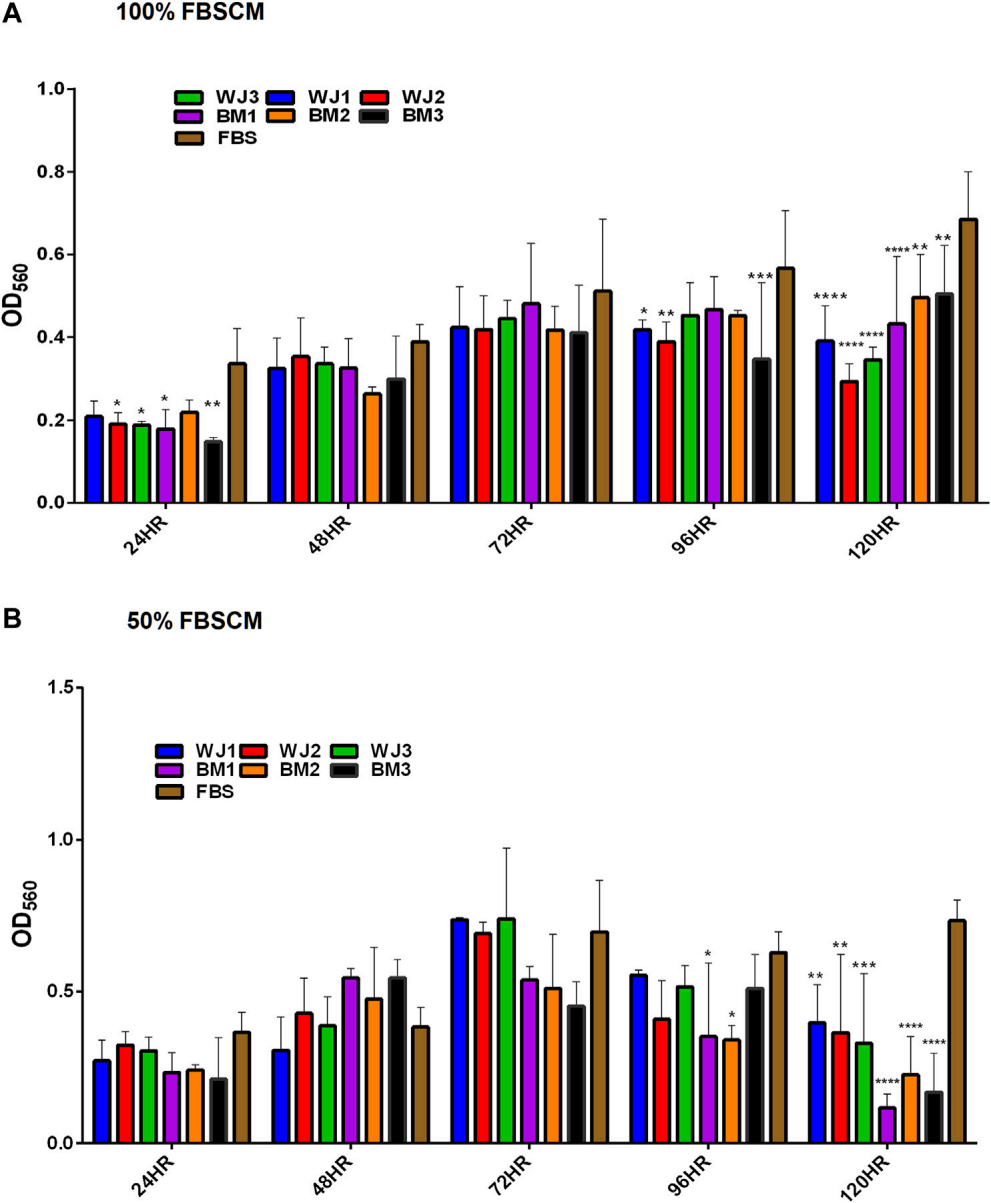
Evaluation of the effect of two concentrations of FBSCM (100%, 50%) on the proliferation of glioma cells (U87MG) at different time points. **(A)** 100% FBSCM. **(B)** 50% FBSCM. The significant inhibitory effect was evident at 96HR and 120HR from both concentrations of FBSCM. Concerning this, some samples showed the inhibitory effect at 96HR signifying individual effects of MSCs while all the samples showed significant inhibition at 120HR (*p < 0.05, **p < 0.01,***p < 0.001,****p < 0.0001) from both concentrations of FBSCM. Interestingly, we found an inhibitory effect at 24HR from 100%FBSCM of WJ2, WJ3, BM1, and BM3 (*p < 0.05). This difference can be explained by the presence of heterogeneous populations at different cell cycle levels in the culture of glioma cells (U87MG).

The effect of two concentrations of SFCM (100% and 50%) on the proliferative ability of the glioma cells is summarized in Figure 5. The inhibitory effect of SFCM (100% and 50%) started at 96 HR and remained consistent until 120 HR as compared to control SFM (Figures 5A,B). It was noted that some samples started to show a response at 96HR from both concentrations like WJ3 and BM2. However, all biological samples from two types of MSCs showed an inhibitory effect at 120HR with varying degrees of significance irrespective of the concentration as compared to control. Similarly, from FBSCM, we found the likewise inhibitory effect from two types of MSCs with both concentrations (Figures 6A,B) in comparison to FBS control. Interestingly, we found a slightly significant inhibitory effect from 100%FBSCM at 24HR from WJ2, WJ3, BM1, and BM3 which can be explained by the presence of heterogeneous populations at different levels of the cell cycle. Consistent with the effect from SFCM, an inhibitory effect started at 96HR from both concentrations from some samples, while all biological samples from two types of MSCs showed a similar inhibitory effect on the proliferation of glioma cells at 120HR at varying significance levels (Figures 6A,B). The variance in significance level can be explained by the individual biological effect like the age of the donor and heterogeneity within the MSCs populations.

In general, both types of MSCs exhibited statistically significant anti-proliferative effects through conditioned media irrespective of the concentrations in comparison to controls.

#### Proliferation of Fibroblasts

**FIGURE 7:**
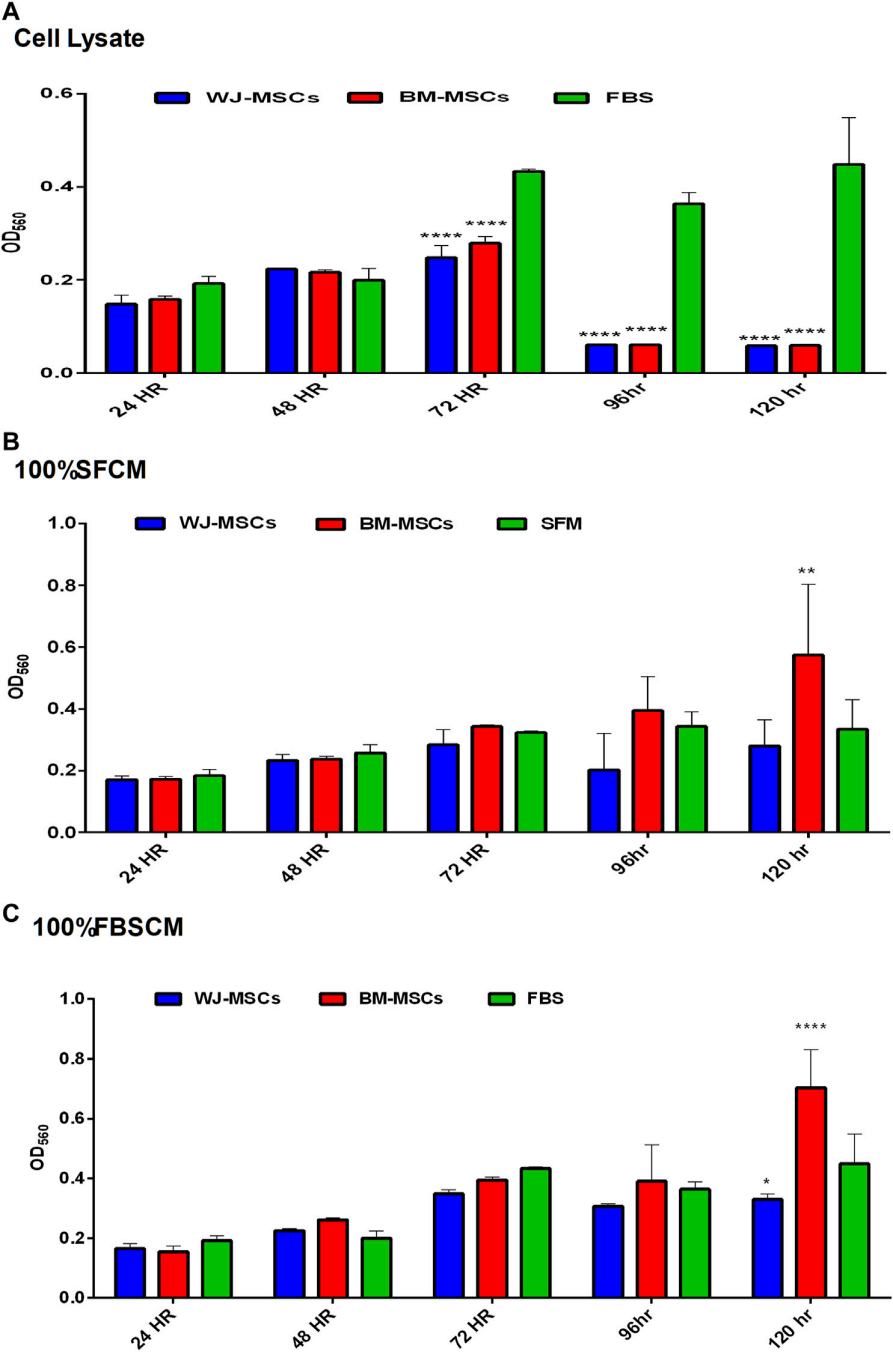
Effect of treatments on the proliferation potential of fibroblasts at different time points. **(A)** Cell lysate. It was noted that cell lysates exerted the same inhibitory effect on the fibroblasts as on glioma cells at 72HR, 96HR, and 120HR (****p < 0.0001). **(B)** (100% SFCM). CM from WJ-MSCs did not induce any stimulatory or inhibitory effect as compared to control SFM at any time point. However, CM from BM-MSCs induced a significant stimulatory effect on the proliferation of fibroblasts as compared to control at 120HR (**p < 0.01). **(C)** (100%FBSCM). BM-MSCs induced a significant stimulatory effect on the proliferation of fibroblasts at 120HR (****p < 0.0001). However, WJ-MSCs exerted a slightly significant inhibitory effect on the proliferation of fibroblasts at 120HR (*p < 0.05) as compared to control FBS.

The effect of all three treatments on the proliferation potential of fibroblasts, from two types of MSCs, is summarized in (Figure 7). The results are presented as averages of WJ-MSCs (WJ1, WJ2, WJ3) and BM-MSCs (BM1, BM2, BM3) from all treatments. It was found that cell lysates significantly inhibited the proliferation of fibroblasts in comparison to FBS control at 72HR, 96HR, and 120HR (Figure 7A). However, we found a variable effect from two types of conditioned media. It was noted that SFCM from BM-MSCs showed a significant increase in the proliferation of fibroblasts as compared to SFM control at 120HR (Figure 7B 100%SFCM) while SFCM from WJ-MSCs lacks any significant stimulatory or inhibitory effect on fibroblasts in comparison to SFM control. Similarly, FBSCM from BM-MSCs showed a significant stimulatory effect on the proliferation of fibroblasts at 120HR as compared to FBS control (Figure 7C 100%FBSCM). However, FBSCM from WJ-MSCs showed a slight but significant inhibitory effect on the proliferation of fibroblasts (Figure 7C). This difference might be due to the interaction of xenogenic proteins of FBS with paracrine factors of MSCs that may have modulated the stimulatory properties of the conditioned media for fibroblasts. In general, cell lysates have the same inhibitory effect on the growth of fibroblasts as on the glioma cells (Figure 7A). SFCM from WJ-MSCs did not show a significant stimulatory or inhibitory effect on the fibroblasts while FBSCM from WJ-MSCs induced an inhibitory effect on the proliferation of fibroblasts. However, both types of CM from BM-MSCs exerted a stimulatory effect on the proliferation of fibroblasts in comparison to two controls**.**

#### Cell Cycle Analysis

**FIGURE 8:**
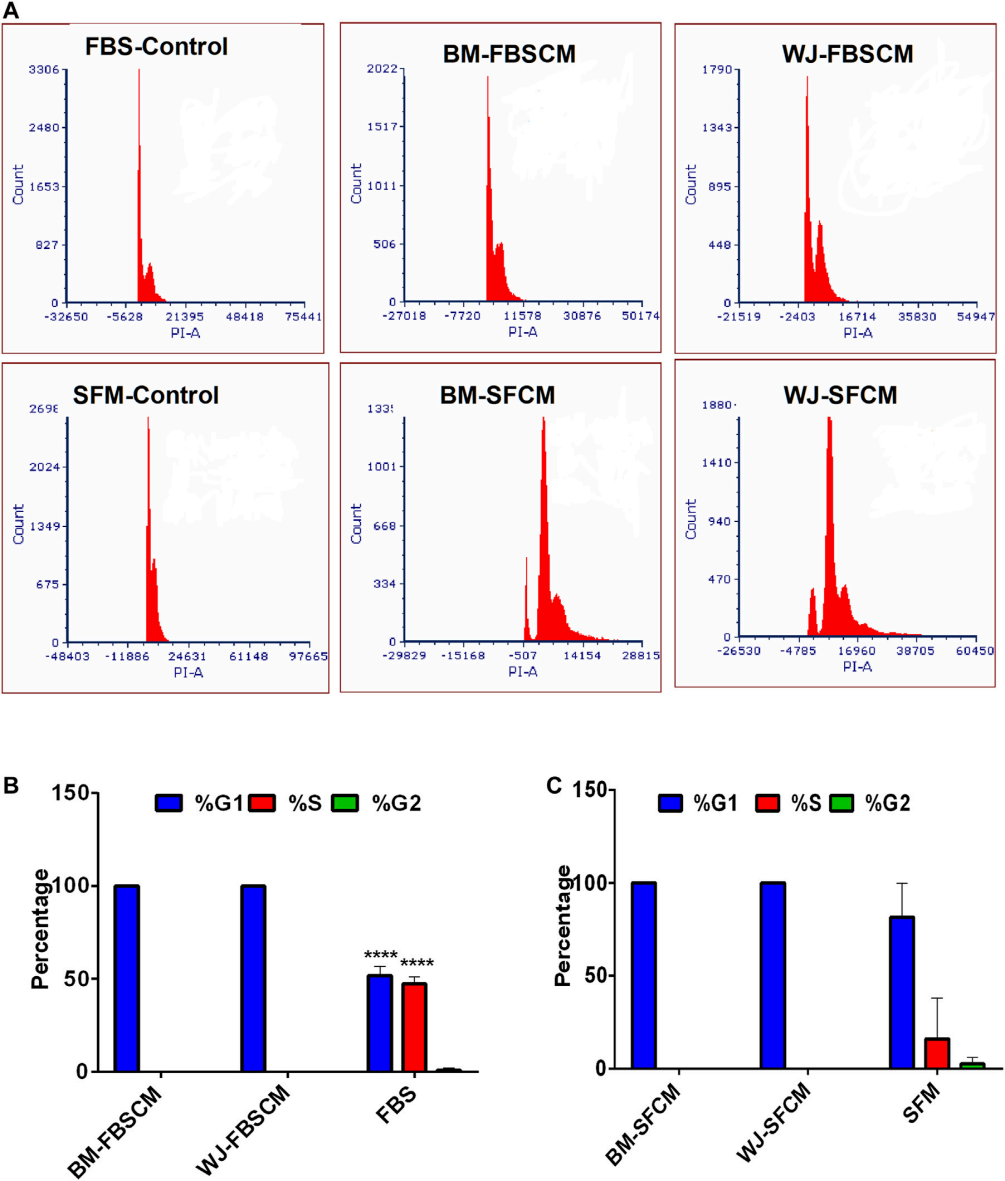
Cell cycle analysis of the glioma cells (U87MG) treated with two types of CM. **(A)** Flow cytometric analysis of the cell cycle of U87MG glioma cells after treating with two types of CM (FBSCM, SFCM) of BM-MSCs and WJ-MSCs for 96HR and stained with propodium iodide (PI). Both types of CM from two types of MSCs showed the growth arrest at the G1 phase as compared to controls. **(B)** Statistical analysis showed significant inhibition at the G1 phase of the cell cycle in treated glioma cells with FBSCM as compared to FBS control (****p < 0.001)*.* **(C)** However, treatment with SFCM did not reach the significance level as compared to SFM control.

Representative images of the results for cell cycle analysis for CM are summarized in Figure 8. It was noted that conditioned media from BM-MSCs (BM1, BM2, BM3 averages) and WJ-MSCs (WJ1, WJ2, WJ3 averages) significantly arrested the glioma cells growth at the G1 phase as compared to FBS and SFM control at 96 HR where 50% of cells already have entered into S phase and 2% in G2. Also in SFM control, 15.9% of cells have already passed into the S phase while 2.5% in G2. From both types of MSCs, we found the growth arrest at the G1 phase with two types of CM as compared to FBS control only while the effect with SFM control was non-significant (Figure 8B averages). This cell cycle arrest may explain the change in morphology and inhibition of the proliferation of the glioma cells.

### Migration Analysis

**FIGURE 9:**
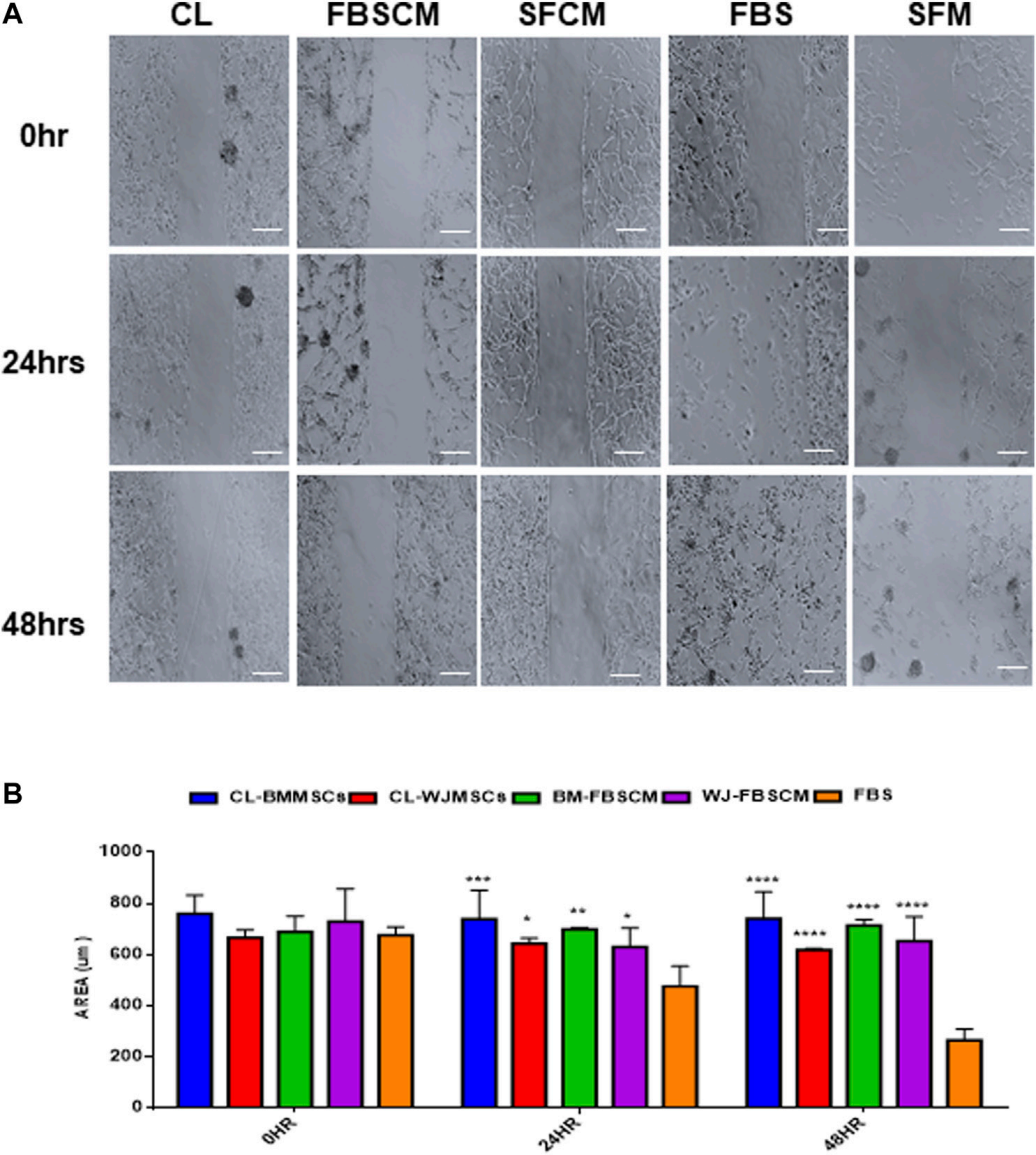
Evaluation of the migration potential of treated glioma cells (U87MG). **(A)** Representative microscopic images of the treated glioma cells with Cell lysate (CL), FBSCM, and SFCM at 0HR, 24HR, and 48HR time points after wound infliction (scale bar, 100 µm). All treatments successfully inhibited the migration potential of glioma cells at 24HR and 48HR. However, glioma cells in SFM control detached at 48HR as a result of serum starvation as shown by sphere formation. **(B)** Statistical analysis showing the significant inhibition of migration potential of glioma cells from CL and FBSCM at 24HR and 48HR as compared to FBS control (*p < 0.05, **p < 0.01, ***p < 0.001,****p < 0.0001). Since the glioma cells in SFM control were detached so the significance levels for SFCM were not evaluated.

The results for migration analysis are summarized in Figure 9. Representative images of the cell migration for 0HR, 24HR, and 48HR have been presented in (Figure 9A). It was noted that all three treatments successfully inhibited the migration capacity of glioma cells at 24HR and 48HR. Statistically, CL and FBSCM from two types of MSCs significantly inhibited the migration potential of the glioma cells in the first 24HR and the effect was continuous till 48HR as compared to FBS control (Figure 9B). Since the glioma cells in SFM control start detaching at 24HR and 48HR as evident by the presence of spheres, therefore, statistical analysis with SFCM could not be evaluated. The detachment of cells in SFM control may be the outcome of the serum starvation step.

### Apoptosis Analysis for Conditioned Media

**FIGURE 10:**
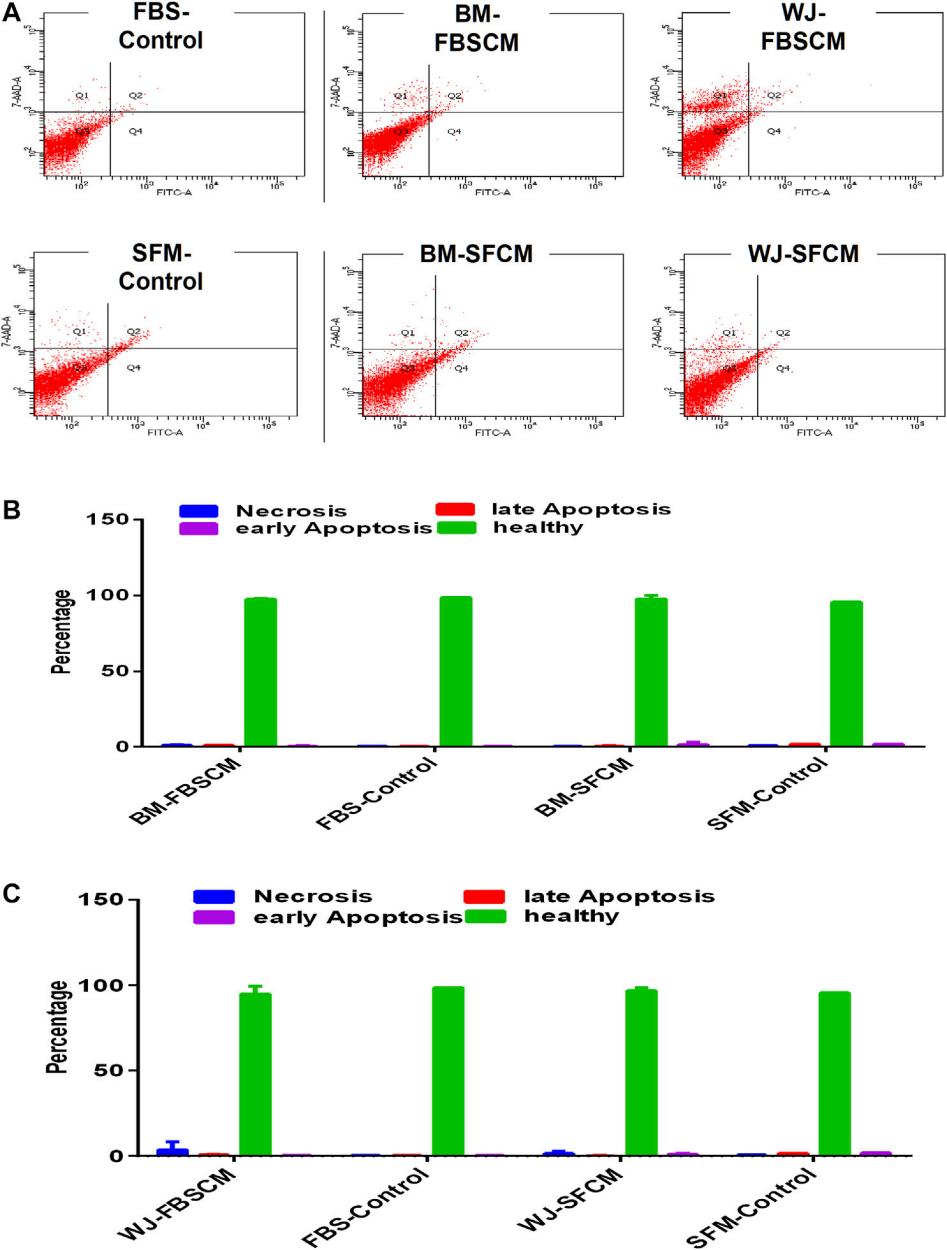
Flow cytometric analysis of the apoptotic effect of two types of conditioned media on the glioma cells (U87MG) stained with AnnexinV/7AAD. **(A)** The representative dot plot is divided into four quadrants; healthy cells (Q3), early apoptotic cells (Q4), necrotic cells (Q1), and late apoptotic cells (Q2). **(B,C)** No significant difference was observed among all treated groups.

From our results, it was clear that cell lysates induced vacuolation in cells at 48HR inducing autophagy or senescence while at 72HR bulk of the glioma cells started degenerating (data not shown). Moreover, cell lysates induced the same inhibitory effect on fibroblasts signifying a generalized inhibitory effect for all types of cells. Therefore, apoptosis analysis was performed on cells treated with CM only. The results for apoptosis analysis are summarized in Figure 10. It was noted that both types of CM did not induce any kind of apoptosis from any type of MSCs. We have noticed slight necrosis with WJ-CM but the effect was not significant. Glioma cells might likely have gone in cell-cycle quiescence at G1 phase due to the inherent ability of stemness since cancer cells have the plasticity to modify themselves according to the change in the environment.

## Discussion

Mesenchymal stem cells (MSCs) could be the next candidate being considered for the treatment of cancer due to the properties of tropism for solid tumors, immune-privileged nature, ease of availability and maintenance, etc [50]. However, the application of MSCs for the possible anti-cancer treatment has divided the scientific society into two groups. One group suggested that MSCs play a crucial role in tumorigenesis by promoting angiogenesis while the other group demonstrated the anti-tumoral effect of MSCs in a few specific cancers [11]. Though, the exact mechanisms of the anti-tumoral property of MSCs are yet to be understood. It has been suggested that the source of MSCs seems to contribute to antitumor properties against particular malignancy. The anti-tumoral effect of umbilical cord blood-derived MSCs was evident against a brain tumor but the same effect was missing with MSCs derived from other tissues [51]. Few studies have reported the particular homing ability of BM-MSCs to tumors that enhanced the metastasis by promoting the migration and angiogenesis and inhibiting apoptosis [52–55]. Many studies also reported that the actual outcome of MSCs' beneficial effects is attributed to the paracrine activities of these cells in the form of cell extracts [21, 22]. To investigate further if the anti-tumoral or pro-tumoral effect of MSCs is subjective to the type of MSCs and whether the cell extracts generated under the same culture conditions may exhibit the same anti-tumoral or pro-tumoral effect, we planned this study.

Regarding morphology, proliferation, and migration potential, our results from cell lysates are following the studies where cells went into autophagy or senescence [3, 44] but we also found the same inhibitory effect of cell lysates on fibroblasts. This is in contradiction to these reports [57, 58]. Perhaps this difference of effect can be attributed to different fibroblast cell lines or the concentrations of cell lysates used in these studies. Recent advancement in the therapeutic potential of cancer vaccines to induce antitumor immune effect caused by cell lysates of cancer cells has opened a novel research horizon [56]. In doing so, this is the first report of the use of cell lysates from MSCs to target glioma cells since glioma patients have a very poor prognosis. However, this area requires further in-depth investigations to rule out specific cytokines, chemokines, or growth factors suitable to cause antitumor immune effect without affecting the normal cells.

About phenotypic results from two types of CM, the treatment did not induce any vacuolation or fragmentation in the glioma cells which conflicts with the previous studies (57.58). Rather we found expanded shapes of cells and a decline in neurite growths. This shows that CM may have induced differentiation or senescence by modulating the microenvironment of the glioma cells and it is supported by our results of the cell cycle analysis where CM treated cells arrested the growth at the G1 phase while control cells were passed into S and G2 phases (Figure 7A).

From the results of the proliferation of the glioma cells, we found significant inhibition from two types of CM (FBSCM and SFCM) at 96HR and 120HR (Figures 5, 6). For WJ-FBSCM, we also found an inhibitory effect at 24HR from some samples. This initial decrease can be explained by the presence of heterogeneous cells at different phases of the cell cycle and the cell's response to arrest or die or proliferate in response to the treatment [57]. Both concentrations of BM-FBSCM and WJ-FBSCM (100% and 50%) showed significant inhibition in comparison to FBS control at 96HR and 120HR. This shows that paracrine factors collected in the presence of FBS can exert inhibitory effect without being modulated by the serum proteins. Though FBS provides a cheap and easily accessible source for essential growth factors and other proteins for the propagation of different types of cells [58]. From a clinical application point of view for cell-free therapy, the collection of paracrine factors in the presence of xenogenic compounds might not be the right choice since immunological reactions have been reported in patients due to the internalization of xenogenic proteins [59]. In this regard, SFCM can be a safe choice for further *in vivo* research since MSC-CM has already been implicated in the treatment of neurodegenerative diseases [42, 43].

On the other hand, the effect of both CM from BM-MSCs on the fibroblasts was stimulatory while FBSCM from WJ-MSCs induced a slight inhibitory effect. It was evident that CM from two types of MSCs in serum-free conditions holds a specific inhibitory effect for glioma cells only. Fibroblasts are an integral part of the tumor microenvironment where they secrete an extracellular matrix (ECM) providing a natural barrier against tumor progression [16, 60]. Contrarily, the same ECM can promote tumor progression and metastasis by modifications caused by secreted proteins of fibroblasts [61]. In this context, the role of cell lysates and CM can be fundamental and need further evaluation in terms of creating an inhibitory effect for the glioma cells only.

In general, we found significant inhibition of migration potential and cells’ metabolic activity (MTT) from all three treatments (CL, SFCM, FBSCM) of two types of MSCs on U87MG glioma cells. Our results are following the previous studies for the anti-tumoral effect from WJ-MSCs [62–67] except for apoptosis. However, our results from BM-MSCs are in contradiction to some earlier studies [13, 52, 67]. Perhaps the reason for this contradiction of biological difference lies with the heterogeneity of MSCs from donors which can be attributed to a diverse repertoire of sub-populations both intra-population or within the same aspirate [68]. Many anti-cancer therapies are targeting cell cycle checkpoints arrest using different modalities. Cell cycle arrest was first identified by lower concentrations of HDAC inhibitors that induced G1 arrest by blocking deacetylase activity in tumors [69]. Many enzymes related to cyclin-dependent kinases (Cdks) are responsible for the correct ordering of cell cycle and progression through the cell cycle is controlled by reversible Cdk phosphorylation [69]. However, Cdk1 alone appears to be the major contributor to cell cycle progression as it can bind to all cyclins [70, 71]. We propose that cell extracts of both MSCs might have arrested the cell cycle by interrupting the pathways related to reversible phosphorylation of Cdk proteins. Cell cycle arrest leads to alter many mechanisms like proliferation, migration, and induction of senescence and differentiation. That might be the reason for the change in morphology, inhibition of proliferation, and inhibition of the migration ability of the glioma cells in our results. However, we did not find any apoptosis as a result of cell cycle arrest which can be explained by the fact that glioma cells might have exhibited cell-cycle quiescence due to the inherent ability of stemness [72].

Unregulated proliferation without stimulation from exogenous mitogens has been the hallmark of cancer which is mediated by the presence of cancer stem cells [73]. It has been reported that glioma CSC/CS-LCs can form spheres in serum-free media without mitogens such as FBS [34, 74]. The short period of serum deprivation can lead to the enrichment of CSCs as a side population (spheres) in cell lines [75, 76]. In our study, sphere formation was quite evident in SFM control as shown by morphology (Figure 4). However, SFCM seems to inhibit the sphere formation in comparison to SFM control (Figure 4). Besides, FBSCM and CL also inhibited the sphere formation in comparison to FBS control (Figures 3A, 4) This shows that cell lysates and conditioned media could have modified the sphere-forming ability of glioma CSCs. However, the functional assay for CSCs along with molecular evaluation (*in vivo*) is required to further strengthen this concept.

## Conclusion

We conclude that cell extracts from two types of MSCs (BM and WJ) successfully induced the inhibition of growth and migration potential of U87MG glioma cells by arresting the cell cycle at the G1 phase. We also present serum-free conditioned media as a preferred source for the collection of paracrine factors since its effect was persistent on the glioma cells from both types of MSCs. Moreover, serum-free media with only a small fraction of exogenous mitogens (1–2%FBS) may provide an important experimental model to investigate the regulation and maintenance of stemness properties of glioma cells in the 2D system (monolayer).

## Data Availability

The raw data supporting the conclusions of this article will be made available by the authors, without undue reservation.

## References

[1] Brat DJ Scheithauer BW Fuller GN Tihan T Newly codified glial neoplasms of the 2007 WHO classification of tumours of the central nervous system: angiocentric glioma, pilomyxoid astrocytoma and pituicytoma. Brain Pathol (2007). 17(3):319–24. 10.1111/j.1750-3639.2007.00082.x 17598825 PMC8095654

[2] Stupp R Hegi ME Mason WP van den Bent MJ Taphoorn MJ Janzer RC Effects of radiotherapy with concomitant and adjuvant temozolomide versus radiotherapy alone on survival in glioblastoma in a randomised phase III study: 5-year analysis of the EORTC-NCIC trial. Lancet Oncol (2009). 10(5):459–66. 10.1016/s1470-2045(09)70025-7 19269895

[3] Gauthaman K Fong C-Y Arularasu S Subramanian A Biswas A Choolani M Human Wharton's Jelly stem cell conditioned medium and cell-free lysate inhibit human osteosarcoma and mammary carcinoma cell growth *in vitro* and in xenograft mice. J Cel Biochem (2013). 114(2):366–77. 10.1002/jcb.24367 22930595

[4] Chamberlain G Fox J Ashton B Middleton J Concise review: mesenchymal stem cells: their phenotype, differentiation capacity, immunological features, and potential for homing. Stem cells (2007). 25 (11), 2739–49. 10.1634/stemcells.2007-0197 17656645

[5] Miranda CO Teixeira CA Liz MA Sousa VF Franquinho F Forte G Systemic delivery of bone marrow-derived mesenchymal stromal cells diminishes neuropathology in a mouse model of krabbe's disease. Stem Cells (2011). 29(11):1738–51. 10.1002/stem.724 21898691

[6] Kidd S Spaeth E Dembinski JL Dietrich M Watson K Klopp A Direct evidence of mesenchymal stem cell tropism for tumor and wounding microenvironments using *in vivo* bioluminescent imaging. Stem cells (2009). 27(10):2614–23. 10.1002/stem.187 19650040 PMC4160730

[7] Kidd S Caldwell L Dietrich M Samudio I Spaeth EL Watson K Mesenchymal stromal cells alone or expressing interferon-β suppress pancreatic tumors *in vivo*, an effect countered by anti-inflammatory treatment. Cytotherapy (2010). 12(5):615–25. 10.3109/14653241003631815 20230221

[8] Loebinger MR Kyrtatos PG Turmaine M Price AN Pankhurst Q Lythgoe MF Magnetic resonance imaging of mesenchymal stem cells homing to pulmonary metastases using biocompatible magnetic nanoparticles. Cancer Res (2009). 69(23):8862–67. 10.1158/0008-5472.can-09-1912 19920196 PMC2833408

[9] Sasportas LS Kasmieh R Wakimoto H Hingtgen S van de Water JAJM Mohapatra G Assessment of therapeutic efficacy and fate of engineered human mesenchymal stem cells for cancer therapy. Proc Natl Acad Sci (2009). 106(12):4822–27. 10.1073/pnas.0806647106 19264968 PMC2660771

[10] Yang B Wu X Mao Y Bao W Gao L Zhou P Dual-targeted antitumor effects against brainstem glioma by intravenous delivery of tumor necrosis factor-related, apoptosis-inducing, ligand-engineered human mesenchymal stem cells. Neurosurgery (2009). 65(3):610–24. 10.1227/01.neu.0000350227.61132.a7 19687708

[11] Roorda BD ter Elst A Kamps WA de Bont ESJM Bone marrow-derived cells and tumor growth: contribution of bone marrow-derived cells to tumor micro-environments with special focus on mesenchymal stem cells. Crit Rev oncology/hematology (2009). 69(3):187–98. 10.1016/j.critrevonc.2008.06.004 18675551

[12] Choumerianou DM Dimitriou H Perdikogianni C Martimianaki G Riminucci M Kalmanti M Study of oncogenic transformation in *ex vivo* expanded mesenchymal cells, from paediatric bone marrow. Cel Prolif (2008). 41(6):909–22. 10.1111/j.1365-2184.2008.00559.x PMC6496314 19040569

[13] Zhang L Xiang J Li G The uncertain role of unmodified mesenchymal stem cells in tumor progression: what master switch?. Stem Cel Res Ther (2013). 4(2):22. 10.1186/scrt170 PMC3707017 23510751

[14] Bartosh TJ Ullah M Zeitouni S Beaver J Prockop DJ Cancer cells enter dormancy after cannibalizing mesenchymal stem/stromal cells (MSCs). Proc Natl Acad Sci USA (2016). 113(42):E6447–E6456. 10.1073/pnas.1612290113 27698134 PMC5081643

[15] Feng B Chen L Review of mesenchymal stem cells and tumors: executioner or coconspirator?. Cancer Biother Radiopharm (2009). 24(6):717–21. 10.1089/cbr.2009.0652 20025552

[16] Maffini MV Soto AM Calabro JM Ucci AA Sonnenschein C The stroma as a crucial target in rat mammary gland carcinogenesis. J Cel Sci (2004). 117(8):1495–502. 10.1242/jcs.01000 14996910

[17] Dezawa M Kanno H Hoshino M Cho H Matsumoto N Itokazu Y Specific induction of neuronal cells from bone marrow stromal cells and application for autologous transplantation. J Clin Invest (2004). 113(12):1701–10. 10.1172/jci200420935 15199405 PMC420509

[18] Wislet-Gendebien S Hans G Leprince P Rigo JM Moonen G Rogister B Plasticity of cultured mesenchymal stem cells: switch from nestin-positive to excitable neuron-like phenotype. Stem cells (2005). 23(3):392–402. 10.1634/stemcells.2004-0149 15749934

[19] Keilhoff G Stang F Goihl A Wolf G Fansa H Transdifferentiated mesenchymal stem cells as alternative therapy in supporting nerve regeneration and myelination. Cell Mol Neurobiol (2006). 26(7-8):1233–50. 10.1007/s10571-006-9029-9 PMC11881818 16779672

[20] Horwitz E Dominici M How do mesenchymal stromal cells exert their therapeutic benefit?. Cytotherapy (2008). 10 (8), 771–4. 10.1080/14653240802618085 19089685

[21] Rosario CM Yandava BD Kosaras B Zurakowski D Sidman RL Snyder EY Differentiation of engrafted multipotent neural progenitors towards replacement of missing granule neurons in meander tail cerebellum may help determine the locus of mutant gene action. Development (1997). 124(21):4213–(24.) 9334270 10.1242/dev.124.21.4213

[22] Li T-S Takahashi M Ohshima M Qin S-L Kubo M Muramatsu K Myocardial repair achieved by the intramyocardial implantation of adult cardiomyocytes in combination with bone marrow cells. Cel Transpl (2008). 17(6):695–703. 10.3727/096368908786092702 18819257

[23] Yang D Wang W Li L Peng Y Chen P Huang H The relative contribution of paracine effect versus direct differentiation on adipose-derived stem cell transplantation mediated cardiac repair. PloS one (2013). 8(3):e59020. 10.1371/journal.pone.0059020 23527076 PMC3602597

[24] Kim HO Choi S-M Kim H-S Mesenchymal stem cell-derived secretome and microvesicles as a cell-free therapeutics for neurodegenerative disorders. Tissue Eng Regen Med (2013). 10(3):93–101. 10.1007/s13770-013-0010-7

[25] Ramdasi S Sarang S Viswanathan C Potential of mesenchymal stem cell based application in cancer. Int J Hematol Oncol Stem Cel Res (2015). 9(2):95 PMC4410295 25922650

[26] Bhang SH Lee S Shin J-Y Lee T-J Jang H-K Kim B-S Efficacious and clinically relevant conditioned medium of human adipose-derived stem cells for therapeutic angiogenesis. Mol Ther (2014). 22(4):862–72. 10.1038/mt.2013.301 24413377 PMC3982496

[27] Ho JCY Lai W-H Li M-F Au K-W Yip M-C Wong NLY Reversal of endothelial progenitor cell dysfunction in patients with type 2 diabetes using a conditioned medium of human embryonic stem cell-derived endothelial cells. Diabetes Metab Res Rev (2012). 28(5):462–73. 10.1002/dmrr.2304 22492468

[28] Di Santo S Yang Z von Ballmoos MW Voelzmann J Diehm N Baumgartner I Novel cell-free strategy for therapeutic angiogenesis: *in vitro* generated conditioned medium can replace progenitor cell transplantation. PloS one (2009). 4(5):e5643. 10.1371/journal.pone.0005643 19479066 PMC2682571

[29] Mirabella T Cilli M Carlone S Cancedda R Gentili C Amniotic liquid derived stem cells as reservoir of secreted angiogenic factors capable of stimulating neo-arteriogenesis in an ischemic model. Biomaterials (2011). 32(15):3689–99. 10.1016/j.biomaterials.2011.01.071 21371750

[30] Kim J Lee JH Yeo SM Chung HM Chae J-I Stem cell recruitment factors secreted from cord blood-derived stem cells that are not secreted from mature endothelial cells enhance wound healing. In Vitro Cell.Dev.Biol.-Animal (2014). 50(2):146–54. 10.1007/s11626-013-9687-0 24190329

[31] Lee MJ Kim J Lee KI Shin JM Chae JI Chung HM Enhancement of wound healing by secretory factors of endothelial precursor cells derived from human embryonic stem cells. Cytotherapy (2011). 13(2):165–78. 10.3109/14653249.2010.512632 21235296

[32] Parekkadan B Van Poll D Suganuma K Carter EA Berthiaume F Tilles AW Mesenchymal stem cell-derived molecules reverse fulminant hepatic failure. PloS one (2007). 2(9):e941. 10.1371/journal.pone.0000941 17895982 PMC1978513

[33] Cantinieaux D Quertainmont R Blacher S Rossi L Wanet T Noël A Conditioned medium from bone marrow-derived mesenchymal stem cells improves recovery after spinal cord injury in rats: an original strategy to avoid cell transplantation. PloS one (2013). 8(8):e69515. 10.1371/journal.pone.0069515 24013448 PMC3754952

[34] Rafiee MR Malekzadeh Shafaroudi A Rohban S Khayatzadeh H Kalhor HR Mowla SJ Enrichment of a rare subpopulation of miR-302-expressing glioma cells by serum deprivation. Cell J (2015). 16(4):494-505. 10.22074/cellj.2015.495 25685740 PMC4297488

[35] Zhao W Ren G Zhang L Zhang Z Liu J Kuang P Efficacy of mesenchymal stem cells derived from human adipose tissue in inhibition of hepatocellular carcinoma cells *in vitro* . Cancer Biother Radiopharm (2012). 27(9):606–13. 10.1089/cbr.2011.1150 22917212

[36] Ryu H Oh J-E Rhee K-J Baik SK Kim J Kang SJ Adipose tissue-derived mesenchymal stem cells cultured at high density express IFN-β and suppress the growth of MCF-7 human breast cancer cells. Cancer Lett (2014). 352(2):220–7. 10.1016/j.canlet.2014.06.018 25016057

[37] Takahara K Ii M Inamoto T Komura K Ibuki N Minami K Adipose-derived stromal cells inhibit prostate cancer cell proliferation inducing apoptosis. Biochem biophysical Res Commun (2014). 446(4):1102–7. 10.1016/j.bbrc.2014.03.080 24680678

[38] Ahn JO Coh YR Lee HW Shin IS Kang SK Youn HY Human adipose tissue-derived mesenchymal stem cells inhibit melanoma growth *in vitro* and *in vivo* . Anticancer Res (2015). 35(1):159–(68.) 25550547

[39] Böhrnsen F Fricke M Sander C Leha A Schliephake H Kramer FJ Interactions of human MSC with head and neck squamous cell carcinoma cell line PCI-13 reduce markers of epithelia-mesenchymal transition. Clin Oral Invest (2015). 19(5):1121–28. 10.1007/s00784-014-1338-7 25346374

[40] Secchiero P Zorzet S Tripodo C Corallini F Melloni E Caruso L Human bone marrow mesenchymal stem cells display anti-cancer activity in SCID mice bearing disseminated non-Hodgkin's lymphoma xenografts. PloS one (2010). 5(6):e11140. 10.1371/journal.pone.0011140 20585401 PMC2886845

[41] Wieder H Beer AJ Holzapfel K Henninger M Maurer T Schwarzenboeck S 11C-choline PET/CT and whole-body MRI including diffusion-weighted imaging for patients with recurrent prostate cancer. Oncotarget (2017). 8(39):66516. 10.18632/oncotarget.16227 29029532 PMC5630432

[42] Mehrabadi S Sadr SS Hoseini M Stem cell conditioned medium as a novel treatment for neuroinflamation diseases. Int J Med Invest (2019). 8(3):1–(12.).

[43] Dahbour S Jamali F Alhattab D Al-Radaideh A Ababneh O Al-Ryalat N Mesenchymal stem cells and conditioned media in the treatment of multiple sclerosis patients: clinical, ophthalmological and radiological assessments of safety and efficacy. CNS Neurosci Ther (2017). 23(11):866–74. 10.1111/cns.12759 28961381 PMC5698713

[44] Gauthaman K Fong C-Y Suganya C-A Subramanian A Biswas A Choolani M Extra-embryonic human Wharton's jelly stem cells do not induce tumorigenesis, unlike human embryonic stem cells. Reprod Biomed Online (2012). 24(2):235–46. 10.1016/j.rbmo.2011.10.007 22196893

[45] Bajetto A Pattarozzi A Corsaro A Barbieri F Daga A Bosio A Different effects of human umbilical cord mesenchymal stem cells on glioblastoma stem cells by direct cell interaction or via released soluble factors. Front Cell Neurosci (2017). 11:312. 10.3389/fncel.2017.00312 29081734 PMC5645520

[46] Pavon LF Marti LC Sibov TT Malheiros SM Brandt RA Cavalheiro S *In vitro* analysis of neurospheres derived from glioblastoma primary culture: a novel methodology paradigm. Front Neurol (2014). 4:214. 10.3389/fneur.2013.00214 24432012 PMC3883037

[47] Wei X Yang X Han Z-p. Qu F-f. Shao L Shi Y-f. Mesenchymal stem cells: a new trend for cell therapy. Acta Pharmacol Sin (2013). 34(6):747. 10.1038/aps.2013.50 23736003 PMC4002895

[48] Prockop DJ Oh JY Medical therapies with adult stem/progenitor cells (MSCs): a backward journey from dramatic results *in vivo* to the cellular and molecular explanations. J Cel Biochem (2012). 113(5):1460–1469. 10.1002/jcb.24046 PMC4147853 22213121

[49] Russell KC Phinney DG Lacey MR Barrilleaux BL Meyertholen KE O'Connor KC . *In Vitro* high-capacity assay to quantify the clonal heterogeneity in trilineage potential of mesenchymal stem cells reveals a complex hierarchy of lineage commitment. Stem cells (2010). 28(4):788–98. 10.1002/stem.312 20127798

[50] Baglio SR Pegtel DM Baldini N Mesenchymal stem cell secreted vesicles provide novel opportunities in (stem) cell-free therapy. Front Physiol (2012). 3:359. 10.3389/fphys.2012.00359 22973239 PMC3434369

[51] Yu JM Jun ES Bae YC Jung JS Mesenchymal stem cells derived from human adipose tissues favor tumor cell growth *in vivo* . Stem Cell Dev (2008). 17(3):463–74. 10.1089/scd.2007.0181 18522494

[52] Studeny M Marini FC Champlin RE Zompetta C Fidler IJ Andreeff M Bone marrow-derived mesenchymal stem cells as vehicles for interferon-beta delivery into tumors. Cancer Res (2002). 62(13):3603–(8.). 12097260

[53] Mishra PJ Mishra PJ Humeniuk R Medina DJ Alexe G Mesirov JP Carcinoma-Associated fibroblast-like differentiation of human mesenchymal stem cells. Cancer Res (2008). 68(11):4331–9. 10.1158/0008-5472.can-08-0943 18519693 PMC2725025

[54] Spaeth EL Dembinski JL Sasser AK Watson K Klopp A Hall B Mesenchymal stem cell transition to tumor-associated fibroblasts contributes to fibrovascular network expansion and tumor progression. PloS one (2009). 4(4):e4992. 10.1371/journal.pone.0004992 19352430 PMC2661372

[55] Shinagawa K Kitadai Y Tanaka M Sumida T Kodama M Higashi Y Mesenchymal stem cells enhance growth and metastasis of colon cancer. Int J Cancer (2010). 127(10):2323–33. 10.1002/ijc.25440 20473928

[56] Gleisner MA Pereda C Tittarelli A Navarrete M Fuentes C Ávalos I A heat-shocked melanoma cell lysate vaccine enhances tumor infiltration by prototypic effector T cells inhibiting tumor growth. J Immunother Cancer (2020). 8(2):e000999. 10.1136/jitc-2020-000999 32690772 PMC7373330

[57] Granada AE Jiménez A Stewart-Ornstein J Blüthgen N Reber S Jambhekar A The effects of proliferation status and cell cycle phase on the responses of single cells to chemotherapy. MBoC (2020). 31(8):845–57. 10.1091/mbc.e19-09-0515 32049575 PMC7185964

[58] Healy L Hunt C Young L Stacey G The UK Stem Cell Bank: its role as a public research resource centre providing access to well-characterised seed stocks of human stem cell lines. Adv Drug Deliv Rev (2005). 57(13):1981–8. 10.1016/j.addr.2005.07.019 16290151

[59] Spees JL Gregory CA Singh H Tucker HA Peister A Lynch PJ Internalized antigens must be removed to prepare hypoimmunogenic mesenchymal stem cells for cell and gene therapy. Mol Ther (2004). 9(5):747–56. 10.1016/j.ymthe.2004.02.012 15120336

[60] Kunz-Schughart LA Knuechel R Tumor-associated fibroblasts (part II): functional impact on tumor tissue. Histol Histopathol (2002). 17, 623. 10.14670/HH-17.623 11962762

[61] Yamashita M Ogawa T Zhang X Hanamura N Kashikura Y Takamura M Role of stromal myofibroblasts in invasive breast cancer: stromal expression of alpha-smooth muscle actin correlates with worse clinical outcome. Breast cancer (2012). 19(2):170–6. 10.1007/s12282-010-0234-5 20978953

[62] Akimoto K Kimura K Nagano M Takano S To'a Salazar G Yamashita T Umbilical cord blood-derived mesenchymal stem cells inhibit, but adipose tissue-derived mesenchymal stem cells promote, glioblastoma multiforme proliferation. Stem Cell Dev (2012). 22(9):1370–86. 10.1089/scd.2012.0486 PMC3696928 23231075

[63] Kim D-W Staples M Shinozuka K Pantcheva P Kang S-D Borlongan C Wharton's jelly-derived mesenchymal stem cells: phenotypic characterization and optimizing their therapeutic potential for clinical applications. Int. J. Mol. Sci (2013). 14(6):11692–712. 10.3390/ijms140611692 23727936 PMC3709752

[64] Rachakatla RS Marini F Weiss ML Tamura M Troyer D Development of human umbilical cord matrix stem cell-based gene therapy for experimental lung tumors. Cancer Gene Ther (2007). 14(10):828. 10.1038/sj.cgt.7701077 17599089

[65] Chao K-C Yang H-T Chen M-W Human umbilical cord mesenchymal stem cells suppress breast cancer tumourigenesis through direct cell-cell contact and internalization. J Cell Mol Med (2012). 16(8):1803–15. 10.1111/j.1582-4934.2011.01459.x 21973190 PMC3822693

[66] Ma Y Hao X Zhang S Zhang J The *in vitro* and *in vivo* effects of human umbilical cord mesenchymal stem cells on the growth of breast cancer cells. Breast Cancer Res Treat (2012). 133(2):473–85. 10.1007/s10549-011-1774-x 21947651

[67] Fan C-G Zhang Q-j. Zhou J-r. Therapeutic potentials of mesenchymal stem cells derived from human umbilical cord. Stem Cel Rev Rep (2011). 7(1):195–207. 10.1007/s12015-010-9168-8 20676943

[68] Pevsner-Fischer M Levin S Zipori D The origins of mesenchymal stromal cell heterogeneity. Stem Cel Rev Rep (2011). 7(3):560–8. 10.1007/s12015-011-9229-7 21437576

[69] Eckschlager T Plch J Stiborova M Hrabeta J Histone deacetylase inhibitors as anticancer drugs. Int. J. Mol. Sci (2017). 18 (7):1414. 10.3390/ijms18071414 PMC5535906 28671573

[70] Santamaría D Barrière C Cerqueira A Hunt S Tardy C Newton K Cdk1 is sufficient to drive the mammalian cell cycle. Nature (2007). 448:811–5. 10.1038/nature06046 17700700

[71] Gérard C Goldbeter A The balance between cell cycle arrest and cell proliferation: control by the extracellular matrix and by contact inhibition. Interf Focus (2014). 4(3):20130075. 10.1098/rsfs.2013.0075 PMC3996587 24904738

[72] Bruschini S Ciliberto G Mancini R The emerging role of cancer cell plasticity and cell-cycle quiescence in immune escape. Cell Death Dis (2020). 11:471. 10.1038/s41419-020-2669-8 32555172 PMC7303167

[73] Zamarin D Holmgaard RB Subudhi SK Park JS Mansour M Palese P Localized oncolytic virotherapy overcomes systemic tumor resistance to immune checkpoint blockade immunotherapy. Sci translational Med (2014). 6(226):226ra32. 10.1126/scitranslmed.3008095 PMC4106918 24598590

[74] Kelly JJP Stechishin O Chojnacki A Lun X Sun B Senger DL Proliferation of human glioblastoma stem cells occurs independently of exogenous mitogens. Stem cells (2009). 27(8):1722–33. 10.1002/stem.98 19544433

[75] Tavaluc RT Hart LS Dicker DT El-Deiry WS Effects of low confluency, serum starvation and hypoxia on the side population of cancer cell lines. Cell cycle (2007). 6(20):2554–62. 10.4161/cc.6.20.4911 17912032

[76] López-Lázaro M Pastor N Azrak SS Ayuso MJ Austin CA Cortés F Digitoxin inhibits the growth of cancer cell lines at concentrations commonly found in cardiac patients. J Nat Prod (2005). 68(11):1642–5. 10.1021/np050226l 16309315

